# Inaccessibility, unresponsiveness, inconsistency, and invisibility of informal caregivers of older persons with cognitive impairment: community-based participatory research

**DOI:** 10.1186/s12877-023-04542-5

**Published:** 2023-12-06

**Authors:** Tsuyoshi Okamura, Chiaki Ura, Yukiko Kugimiya, Mutsuko Okamura, Masako Yamamura, Hidemi Okado, Mayumi Kaneko, Mari Yamashita, Tomoko Wakui

**Affiliations:** 1 Research Team for Promoting Independence and Mental Health, Tokyo Metropolitan Institute of Gerontology, Tokyo, Japan; 2 Research Team for Human Care, Tokyo Metropolitan Institute of Gerontology, Tokyo, Japan

**Keywords:** Older persons, Family caregiver, Informal caregiver, Caregivers, Japan, Traditional caregiver, Cognitive impairment

## Abstract

**Background:**

Studies on informal caregivers in Japan have been limited to family caregivers and largely conducted where family caregivers generally gather. Family caregivers who do not visit such places or non-family caregivers are generally overlooked, and data on these informal caregivers remains scant. Consequently, a framework is needed through which healthcare professionals can approach the informal caregivers of community-dwelling older persons. Therefore, this study approaches such informal caregivers and proposes a classification system for them from the starting point of older persons living in the community with cognitive impairment.

**Methods:**

In 2016, we conducted an epidemiological survey of 7000 + community-dwelling older persons and identified 198 residents with Mini-mental state examination scores less than 23. A team of healthcare professionals contacted them regularly. By 2022, 92 people were still living in the community, and we systematically asked them about their informal caregivers. After approaching the caregivers and obtaining informed consent, we mailed separate questionnaires to older persons and informal caregivers.

**Results:**

Among the caregivers, 59%, 34%, and 3% were the child, spouse, and sibling of the older person, while the remaining 4% were non-family caregivers. Except for two daughters-in-laws, all children were biological children of the older person. Male caregivers (46%) tended to have full-time jobs, whereas female caregivers (54%) tended to face financial difficulties. Only 3% of the caregivers had joined a family caregivers’ association. Caregivers’ reason for not joining such organizations was a lack of time and knowledge. A 3-tiered classification system was developed for these informal caregivers: (1) the household form, (2) accessibility, and (3) the reciprocal awareness of caregiving. Furthermore, family caregivers who lived with the older person or visited them more than once a week with reciprocal awareness of caring and being cared, or “traditional caregivers,” accounted for 68% of the caregivers in this study.

**Conclusion:**

Core family caregivers can be easily approached at places where such caregivers generally gather. However, there also exists a group of informal caregivers who are sometimes inaccessible, unresponsive, and invisible to healthcare professionals. Moreover, their awareness of caregiving is sometimes inconsistent.

**Supplementary Information:**

The online version contains supplementary material available at 10.1186/s12877-023-04542-5.

## Background

Providing care to older people has been reported to decrease psychological wellbeing [1]. There have been several empirical studies on family caregivers, and they have shown that family caregivers of persons with dementia face emotional and social challenges [2]. Steenfeldt et al. [3] found that the challenges faced by family caregivers living with a person with dementia can be classified into three categories: (1) the caregiver’s new roles and relationships, (2) caregiver burdens, and (3) the lack of information and support. It has also been found that the burden on family caregivers is affected by their relationship with the patient. Family caregivers in families with poor affinity are reported to be more likely to experience increased caregiver burden [4].

Caregiving is affected by cultural, normative, political, institutional, and sex-based factors. In Japan, caring for people with dementia used to be the housewife’s responsibility. However, the introduction of the long-term care insurance (LTCI) drastically changed this situation [5]. After the LTCI’s introduction, caring for a person with dementia was rapidly socialized by creating a new profession called care manager. This change was the result of shrinking and aging Japanese families. Notably, of the households that have a person aged 65 or older, 29% are those where the older person lives alone, 32% are those where only old spouses live, 20% are those where older parent(s) live(s) with their unmarried child(ren), and only 10% are those where three generations or a traditional Japanese family lives [6].

Previous studies on informal caregivers in Japan have been limited to family caregivers. They have explored different perspectives and reported on family caregivers’ burdens. Nagai et al. [7] analyzed family caregiving from the perspective of occupational therapy. They suggested that family caregivers’ emotions, their lives, and the relationship between caregiving and the home environment are important components of family caregiving. From the perspective of home-visiting nurses, Saito and Hatano [8] reported that sex-based differences exist in family caregivers’ distress; lack of self-efficacy and lack of self-esteem was related to depression in male and female caregivers, respectively. While these studies conducted qualitative analyses, Iwata et al. [9] surveyed more than 1,000 caregivers and reported a complex association between family structure and caregivers’ distress. Tsuyuki et al. [10] conducted a longitudinal study to analyze the narratives of family caregivers by participating in and observing family meetings over several years. They found that family caregivers’ psychological process is dynamic and encompasses “recognizing problems,” “seeking help,” “evaluating problems,” and “empowering themselves to overcome problems.”

Generally, it is difficult to find participants for such studies because family caregivers are busy and exhausted. The abovementioned studies adopted reasonable strategies to approach them, such as contacting them at places where they generally gathered, like offices of home-visiting nurses, hospitals, and family caregivers’ associations. Resultingly, the participation of informal caregivers who cannot use such services, come to hospitals, or join caregivers’ associations remains limited in previous studies. Moreover, data on non-family caregivers are sparse. In this study, we use the term “family” to refer to spouses, parents, children, siblings, and parents and children of spouses, in accordance with common Japanese social practice. Thus, it becomes imperative to collect informal caregivers’ basic data in the community itself rather than where they generally gather.

Our clinical experiences also emphasize this need. We have been conducting community-based participatory research (CBPR) in Tokyo for six years, running epidemiological and action research in parallel [11]. We provide professional consultation to community residents at our CBPR center and often meet family caregivers who are not connected to family associations, home-visiting nurses, or sometimes even hospitals [12].

Therefore, this study verifies the clinical knowledge we have obtained in the action research arm of our CBPR through the other arm—epidemiological research —by systematically approaching the informal caregivers of community-dwelling older persons. As stated earlier, the existing frameworks are based on research that is conducted where family caregivers of older persons generally gather. However, often, health care professionals only meet the older persons in clinical settings, not their informal caregivers. This highlights the need to have a new framework through which healthcare professionals can access these informal caregivers. Therefore, we also present a classification of informal caregivers that starts right from the source—the older persons they provide care to. This classification can help healthcare professionals approach informal caregivers of older persons with cognitive impairment in clinical settings.

In this study, we aimed to access and classify the informal caregivers of community-dwelling older persons living with cognitive impairment, as opposed to previous studies that have largely focused on family caregivers. We analyzed the process and outcome of this research-based access to construct a new framework of caregivers of older people with cognitive impairment that may prove useful for the healthcare professionals in clinical settings. In addition, we believe this framework will provide a comprehensive picture of the current situation of informal caregivers through its classification system. However, there is scope for further research with respect to how this new framework will work in a real medical setting.

## Methods

**Fig. 1:**
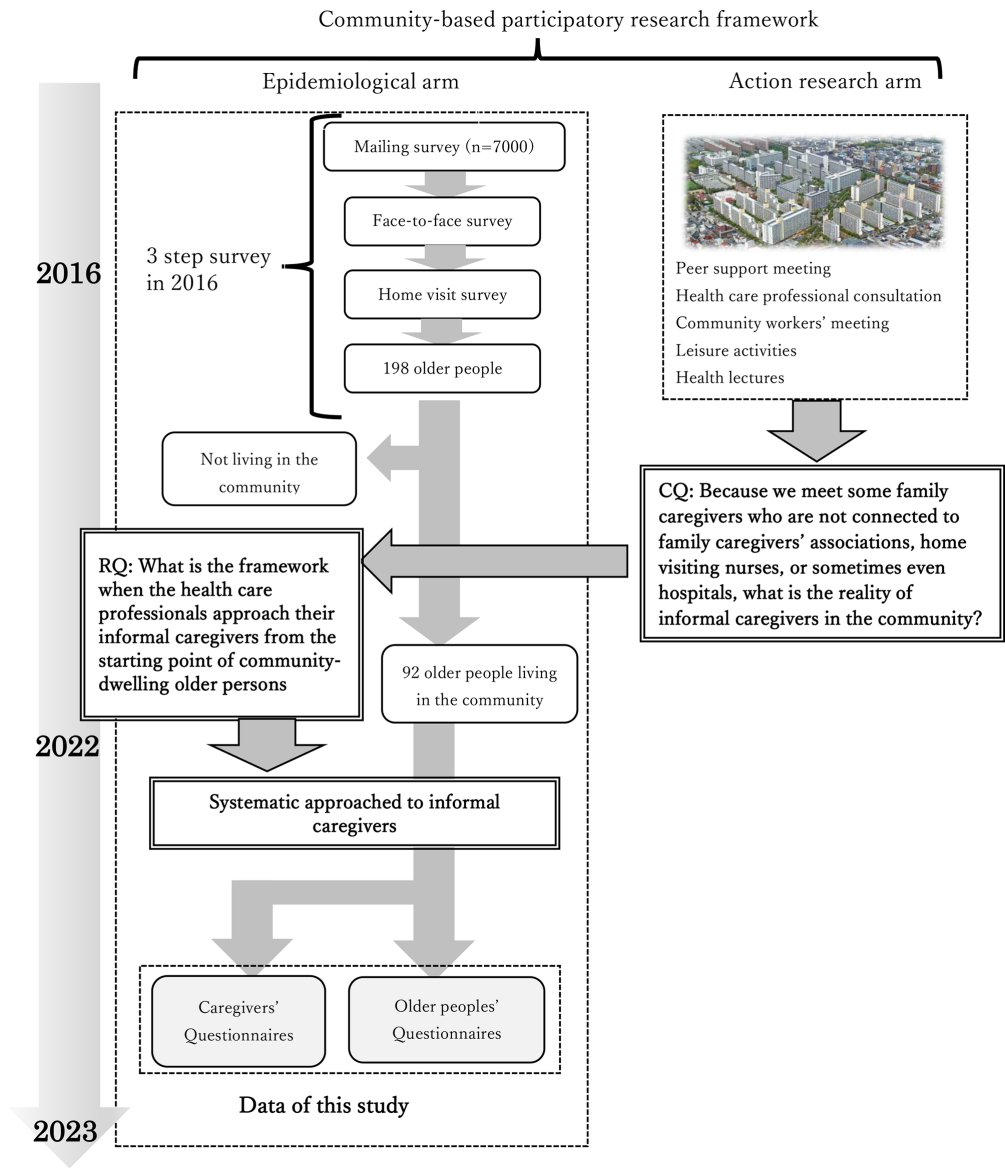
Formation of clinical question and research question which are presented in the flow of the study and the community-based participatory research framework

Figure 1 illustrates the flow and methodology of this study.

### Participants

In 2016, we conducted a three-step survey, which encompassed a mailing survey, a face-to-face survey, and a home-visit survey. The mailing survey was conducted on 7614 older persons living in the study area [13]. In mailing survey, the basic information of the older persons was collected, and an invitation was sent for the subsequent surveys. In the face-to-face survey which were conducted on all of the applicants, we performed a Mini-mental state examination on 2020 participants, and 335 persons who scored 22 or less were subjected to the home-visit survey. Because 137 people did not wish to participate to home-visit survey, 198 people underwent assessments by professionals in their own homes. Since then, they have been contacted annually [14]. After six years, as of July 1, 2022, 92 older persons were still living in the community (46%), including three who were hospitalized. For others, 28 persons died (14%), 30 persons institutionalized (15%), 2 withdrew from participation (1%), or 16 no longer lived there (8%), and 30 with the reasons unknown (15%). These 92 older persons were considered the participants of this study.

### Setting

This study was conducted in a large housing complex and its surrounding area located northwest of the Tokyo metropolitan area. We built a permanent CBPR center at the center of the housing complex. At this center, residents could consult healthcare professionals, such as psychiatrists, psychologists, and public health nurses, who were also the research staff. These consultations were not part of the medical or welfare system; they were provided free of charge [15].

### Process

This study was conducted from September to December 2022.

#### Phase I: preparing for approaching the informal caregivers

Since this was the first time researchers were systematically approaching informal caregivers, a research team comprising psychiatrists, psychologists, and public health nurses created a manual for making phone calls. We encountered various unexpected situations in Phases 2 and 3. Therefore, research meetings were held once a week, and the manual was revised to make it fit for all situations.

#### Phase II: making the telephone calls

First, we sent a letter to participants’ homes to inform them of the study’s aim and method. When a letter was received, a public health nurse made a phone call to the participant. The participant was then asked if they had an informal caregiver. The questions we used were, “Who are the family members, relatives, or persons who take care of you?,” “Do you have a family member, relative, or person who comes to your house to care of you?,” and “Do you have a family member, relative, or person who regularly contacts you because they care about you?” Then, the researchers asked for the informal caregiver’s name, relationship with the older person, sex, contact information, and whether they lived with the older person.

If an informal caregiver lived with the older person, the older person was asked to hand over the phone to the caregiver. We explained the study’s aim and methods to the caregiver and obtained their consent to send a questionnaire. If the informal caregiver did not live with the older person, we telephoned them if the older person provided their contact information. If the older person was not willing to provide the caregiver’s contact information but willing to participate in the survey, we sent the questionnaire to the older person’s home to be filled by the caregiver when they visited the older person’s home.

#### Phase III: mailing the survey

Questionnaires were mailed to both older persons and informal caregivers. If the older person forgot to return the survey after a certain period, a public health nurse or other professional called them and interviewed them over the phone.

### Measures

Since the participants included people with cognitive impairment, we made the questionnaire as simple as possible. The questions used in this study are provided as Additional file 1. These questions were prepared in consultation with the staff at the CBPR center to ensure that our intentions are accurately conveyed. In addition, we were advised to be cautious when asking about respondents’ family members.

In the older persons’ questionnaire, the respondents were asked to describe the “help and assistance their caregiver provided.” Some of the responses included, “I do not receive any help.”

In the caregivers’ questionnaire, respondents were asked to provide basic information about themselves, such as age, sex, socioeconomic status, employment status, relationship with the older person, whether they lived with the older person, and whether they belonged to a family caregivers’ association. If they were not part of a family caregivers’ association, they were asked to provide the reason for it in an open-ended statement. The respondents were also asked about their caregiving, such as the level of care required, the duration and frequency of caregiving, and the details of the care provided.

In summary, we asked about caregiving from both parties. The caregivers were asked to indicate the frequency of caregiving on a scale of “never, ” “sometimes,” and “always,” concerning these activities: helping in bathing, dressing, brushing teeth, elimination, indoor transportation, eating, meal preparation, shopping for daily necessities, cleaning and laundry, accompanying outdoors, taking medicines, managing finances, going to the hospital, coordinating with the care manager, talking, sorting medicines, communicating with relatives and friends, accompanying them when they wander, calming them when they get confused due to cognitive impairment, going to rehabilitation to maintain physical functions, enjoying brain training, adjusting the living environment, managing physical condition, and preparing luggage to go to day-care services.

### Japanese context

We also asked respondents about Japan’s LTCI program. In 2000, Japan introduced the LTCI system for people aged 65 years and above, which covers their care costs and requires the user to pay 10% of the total cost as co-payment. To receive long-term care insurance services, people must be “certified” and are generally certified when they recognize that they need services as their physical and cognitive functions deteriorate. Certification itself is easy to apply for. However, some people do not receive “certification” and receive support and care by family members. Caregiving has traditionally been forced on females. In fact, according to a 2007 household survey of long-term care service in Japan [16], more than 70% of caregivers of people with dementia were female. However, few studies have explored this glaring disparity. We therefore included sex as a study measure. Additionally, socioeconomic status was also included as a measure because the economic difficulties of caregivers of persons with dementia have been previously reported [17].

As for family caregiver organizations, public support has largely been scarce in Japan because caregiving has long been considered a family business. The first family caregivers’ association was established in Kyoto in 1980 by 90 family caregivers on a voluntary basis. This association was established to help people understand dementia, learn how to appropriately care for it, reduce anxiety and stress among caregivers, and to promote understanding of dementia in the community. Since then, family associations have sprung up in various regions, with a total of 10,000 members by 2017 [18]. These associations have also started to work together to create an umbrella organization.

### Data analysis

The data we obtained were: (1) records of telephone calls (structured notes), (2) older persons’ responses, and (3) caregivers’ responses.

First, we attempted to describe the actual situation of informal caregivers in the community using basic information. A χ^2^ test was also conducted to determine sex-based differences in caregivers’ socioeconomic and employment statuses. Second, public health nurses who telephoned older persons and caregivers and the researcher who designed this study reviewed the telephone call phase and discussed the classification of informal caregivers. Then, the researcher discussed the data gathered from a multidisciplinary perspective. The resulting classification was discussed with the public health nurses until a consensus was reached and a final classification was created.

### Ethical considerations

The study protocol was approved by the Ethics Committee of Tokyo Metropolitan Institute of Gerontology (R22-026). This study was conducted in accordance with the code of ethics set by the Declaration of Helsinki and its future amendments or comparable standards.

All participants provided written informed consent.

For additional consent of surrogates of older people, we followed the recommendations of the Global Alliance for Genomics and Health’s Ageing and Dementia Task Team [19]. This recommendation states that researchers should presume that people with dementia have capacity until they demonstrate otherwise. We have been communicating with the participants in this study for seven years. All health professionals involved in the project, including the geriatric psychiatrist, judged that they have a consistent willingness and sufficient capacity to participate in the study on an understanding basis. Therefore, consent was not obtained from legally authorized proxies. In addition, participants in this study were not eligible for guardianship system in Japan. The reason is that, in Japan, legally authorized representatives cannot be carried out unless the person has severe dementia and is unable to protect their property (known as guardianship). The study protocol has been approved by the Ethics Committee of Tokyo Metropolitan Institute of Gerontology as described in this statement.

## Results

### Characteristics of informal caregivers

We received 63 filled-in older person questionnaires and 74 filled-in informal caregiver questionnaires. Of the older person questionnaires, 18 did not have corresponding family caregiver questionnaires. Resultingly, we obtained both-sided information of 45 older persons. Of the family caregiver questionnaires, 14 did not have corresponding older person questionnaires. Of the 94 older persons, we were able to access the informal caregivers of and obtain data from 54 (55%) of them. There was no statistical difference between these 54 older persons and others concerning basic characteristics.

Table 1 presents the characteristics of informal caregivers in the community. Among the caregivers, 34%, 59%, and 3% were the spouse, child, and sibling of the older person, respectively. The remaining 4% were non-family caregivers. Among the caregivers who were the children of the older persons, two were daughters-in-law, and the rest were biological children. Furthermore, 29% of the caregivers were not certified by the LTCI, 74% had been providing care for more than a year, 35% had full-time jobs, and 28% were experiencing financial difficulties. The proportion of full-time workers among male caregivers (50%) was significantly higher than the proportion among female caregivers (23%) (p = 0.016). Only 3% of caregivers had joined a family caregivers’ association. The three non-family caregivers were not part of any family caregivers’ association. The reason for not joining such associations was a lack of time and knowledge.

**Table 1:** Descriptive characteristics of the caregivers in the community who are approached from the starting point of the older person

Age (year ± SD)		68.5 ± 12.7	
Sex	Male	34	(46%)
	Female	40	(54%)
Living together		45	(61%)
Relationships	Spouse	25	(34%)
	Parents	44	(59%)
	Others 2 siblings 3 non family	5	(7%)
LTCI grade	No certification	21	(29%)
	Support needy 1	3	(4%)
	Support needy 2	7	(10%)
	Care needy 1	14	(19%)
	Care needy 2	9	(12%)
	Care needy 3	6	(8%)
	Care needy 4	1	(1%)
	Care needy 5	5	(7%)
	DK	8	(11%)
Duration of care	< 1 year	13	(17%)
	1 year to 3 years	12	(16%)
	3 years to 5 years	19	(26%)
	3 years to 5 years	14	(19%)
	> 10 years	10	(14%)
	Others	5	(7%)
Frequency of care	< one day/month	21	(28%)
	< one day/week	13	(18%)
	2 days to 6 days /week	9	(12%)
	Every day	30	(41%)
Family association	Belonging at present	2	(3%)
	Belonged in past	3	(4%)
	No belonging	69	(93%)
SES	Difficulty	8	(11%)
	Somewhat difficulty	13	(18%)
	Normal	45	(61%)
	Somewhat affluent	2	(3%)
	Affluent	6	(8%)
Employment	Full-time job	26	(35%)
	Part-time job	13	(18%)
	Quitted due to caregiving	3	(4%)
	Quitted due to other reason	20	(27%)
	Not employed	12	(16%)

Concerning the sex difference, there were 40 female caregivers (54%) and 34 male caregivers (46%). Both the respondents who were members of the family caregivers’ association were male. Two of the three non-family caregivers were also male. The proportion of caregivers’ experiencing financial difficulties among female caregivers (38%) was significantly higher than that among male caregivers (18%) (p = 0.073).

### Classification data

#### Caregivers who live with the older person

The case of cohabitation turned out to be more complicated than expected. First, there was one case in which the older person lived with their family but refused to ask their family to participate, saying that they could not ask for help. However, they willingly filled-in the older person questionnaire. Second, in six cases, the caregivers stated that they were not the caregiver at the time of the first call. However, the questionnaire responses confirmed that they were indeed providing different types of care. Third, there was a case in which the caregiver stated, “I have been taking care of my family member without any support, but I am at my limit, and I am afraid that I will abuse them.” The psychiatrist, public health nurse, and the head of the community comprehensive support center immediately visited the person to tell them about the LTCI. In another case, having no caregivers, an older person asked for help because they had no money and were not going to receive their pension until the next month. We visited this older person also and told them about the LTCI.

In summary, a total of 45 caregivers lived with the older person and participated in the study by sending filled-in questionnaires. Out of the 45 caregivers who lived with the older person and participated in the study, one provided care in emergencies. In six cases, reciprocal awareness of caregiving was missing.

#### Caregivers who do not live with the older person

In cases where the older person was living alone, the process of asking for informal caregivers’ contact information was critical. Two older persons stated that they had no family, relatives, or other persons. Four said that they had families but no caregivers because they did not interact with their family members. Six refused to provide contact information because of their distressed relationship with the caregiver. Furthermore, there were three cases in which we telephoned the family member, but they refused to participate.

Among the caregivers who did not live with the older person but participated in the study, 15 provided care at least once a week, and 11 provided care less than once a week. Among those who provided care less than once a week, there were three cases in which the older person insisted that they were not being cared for, but their caregivers had furnished the details of the care they provided. Furthermore, there were three caregivers who were not family members: two provided care to the spouse of a deceased friend, and one provided care to their mother’s aunt.

In summary, 29 caregivers did not live with the older person but participated in the study. Among the cases in which the caregiver did not live with the older person but provided care at least once a week, reciprocal awareness of caregiving was missing in three.

### Classification of informal caregivers

Based on the data mentioned above, we created a classification for informal caregivers from the starting point—older people living in the community. We believe that informal caregivers can be classified based on three tiers. In the first tier, they can be classified based on the household form—whether they live or do not live with the older person. In the second tier, they can be classified based on their accessibility to the research team—whether the caregivers are accessible and responsive. We could not access many caregivers because of a lack of their information and some did not respond to our approach. These inaccessible and unresponsive informal caregivers dropped out of our study. Finally, in the third tier, informal caregivers can be classified based on the perception of caregiving―whether there was reciprocal awareness of caregiving. In some cases, the awareness of caregiving was not present in the two parties: the caregiver and the older person. Without reciprocal awareness of caregiving, it is impossible for health care professionals to coordinate with informal caregivers about the provision of care, and these “inconsistent” caregivers might be overlooked. In this study, the number of cases in which the older person displayed a lack of awareness was four (three mother–son cases and one mother–daughter case); whereas, the number of the cases in which the caregiver showed a lack of awareness was five (one father–son case, two father–daughter cases, and two wife–husband cases). Thus, these inaccessible, unresponsive, and inconsistent informal caregivers remain invisible to healthcare professionals.

**Fig. 2:**
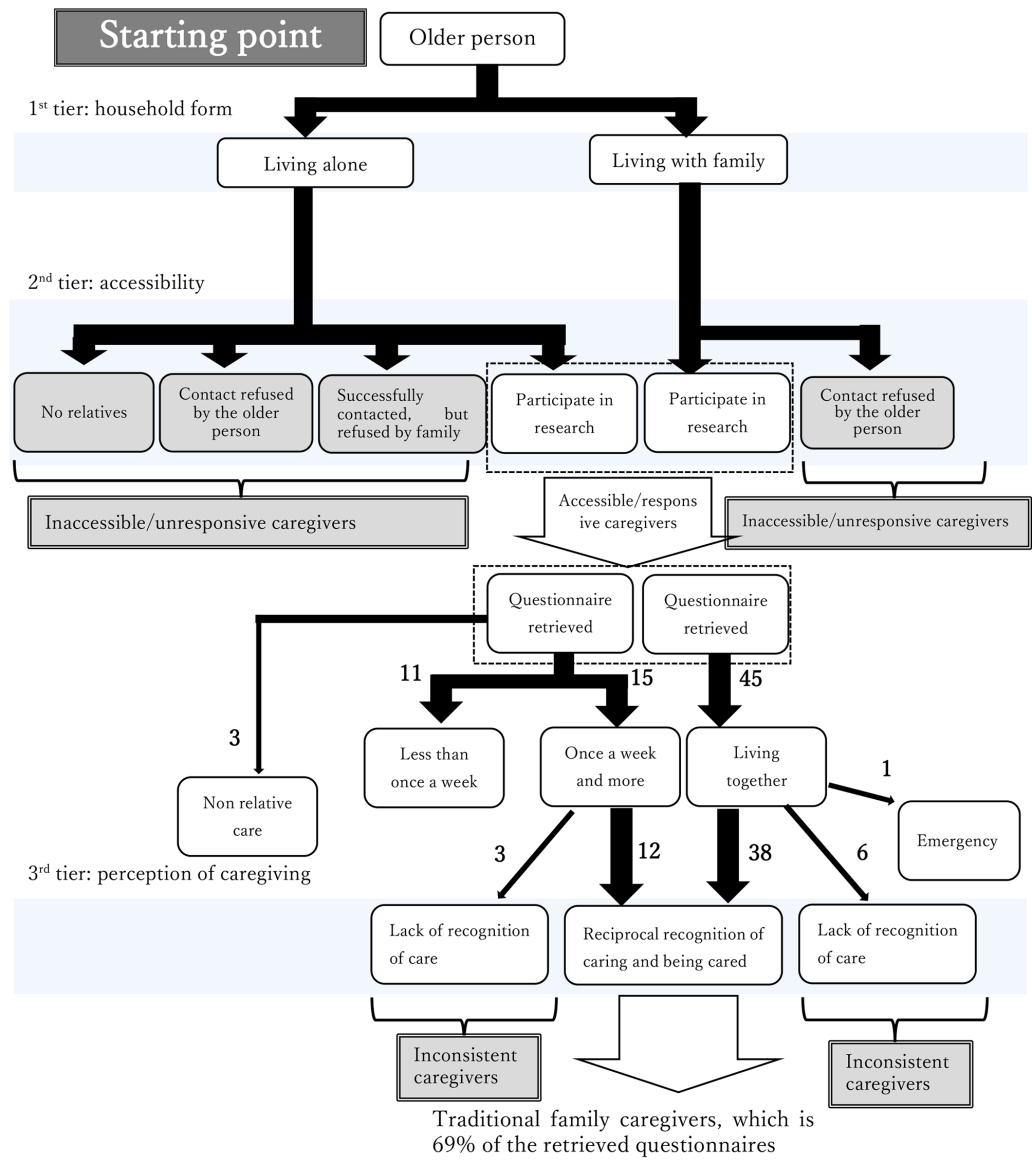
Schema of the access to the informal caregivers from the starting point of older persons

A total of 50 caregivers were family caregivers who either lived with the older person or visited them at least once a week and had reciprocal awareness of caregiving. That is, both parties are aware of the fact that the caregiver is providing care to the older person. Without this awareness, it is impossible for healthcare professionals to coordinate with informal caregivers regarding the care to be provided. We define these caregivers as “traditional caregivers.” They accounted for 68% of the caregivers whose questionnaire responses we had received. Considering that some informal caregivers denied participating, the proportion of traditional caregivers is less than 68%. This schema is illustrated in Fig. 2 with the number of caregiver questionnaires we received.

## Discussion

This study is unique because it approached informal caregivers from the starting point—older persons living in a community under the CBPR scheme. Compared to Sugiura et al.’s [20] study that was conducted in 2002 and showed that the percentage of male caregivers is 26%, we found that there is little difference in the percentage of male and female caregivers in present-day Tokyo, with 46% of the caregivers being male. However, we did find sex-based differences in the challenges caregivers face. Male caregivers tended to have full-time jobs, whereas female caregivers tended to have financial difficulties. We also found that only 3% of the caregivers had joined family caregivers’ associations. This indicates that what previous studies have shown differs significantly from the reality: there also exists a group of caregivers who do not join such associations, and they remain largely unexplored. Considering that caregivers did not join these associations because of a lack of time, it is vital to implement measures that provide sufficient time to caregivers to join family caregivers’ associations. Encouraging caregivers to join such associations is also essential.

Regarding the second tier, caregivers’ accessibility, we were denied access by the older person or their informal caregiver. Since we do not possess data on informal caregivers who were lost at this stage, their details remain unknown. Inaccessibility here can logically mean no more than the inability of the research team to access the relevant data in the current study. However, we also consider the possibility of inaccessibility in actual medical care. We hypothesize that some caregivers or care recipients do not wish for a third party to be involved in the caregiving–care receiving relationship in the real world.

Regarding the third tier, 68% of the caregivers whose questionnaire responses we had received were traditional caregivers. This suggests that in addition to the core family caregivers who can be easily approached in places such as family caregivers’ associations and medical institutions, there is a group of caregivers who do not go to such places but can be approached by the researchers. Except for these caregivers, there also exists a group of caregivers who could not be approached in this study. Based on our clinical experience, these invisible caregivers have certainly been encountered before but have not been systematically studied.

The phenomenon we term inconsistency, wherein the caregiver and the older person do not share the same awareness of caregiving, shows the intrinsic difficulty of taking care of older people with cognitive impairment while respecting their views. If the older person has no awareness of being cared for, they might disagree with social interventions such as LTCI. In this case, the situation may worsen because a caregiver’s respect for the older person’s views would mean that social interventions cannot be implemented. Although caution must be exercised in making generalizations due to the small number of cases analyzed in this study, the fact that the cared-for party in inconsistent cases lacking awareness of “being cared for” on the part of the older person was always mothers, while all inconsistent cases lacking awareness of “caring” on the part of the caregiver were for fathers and wives, may reflect a universal typology of intra-family dynamics that changes with aging. However, an in-depth analysis of this issue is outside the scope of this study.

This study revealed the present status of the caregivers in Japan. Regarding sex differences, although previous research has shown that most caregivers of people with dementia are female, this study found no such difference. However, we did find that more female caregivers faced economic difficulties. Furthermore, only 3% of the respondents were part of a family caregivers’ association. As clinicians, we have experience-based knowledge that a family caregivers’ association helps people in overcoming their caregiving difficulties. However, although this is beyond the scope of the present study, its results suggest the need to explore the benefits and reform of family caregivers’ associations.

This study has several policy implications. We found that informal caregivers are invisible in Japan, a society with an aging population, shrinking families, and an aging-in-place policy. Moreover, the sex ratio of caregivers is changing, with an increasing number of male care providers, and fewer caregivers are participating in family caregivers’ associations, indicating that caregivers are also changing dramatically as society ages. While it is difficult to generalize because of the influence of various cultural and social contexts, this phenomenon of invisible caregivers may be a future occurrence in other societies given that Japan is one of the most aging countries in the world. Based on the findings of this study, we make the following recommendations for a more inclusive society: (1) To understand the actual status of the invisible informal caregivers, it is essential to develop a methodology that does not rely on specific venues, such as family caregivers’ associations, to collect basic data about caregiving; (2) There is a need to build a consensus on acceptable practices when an older person is moved to a medical setting without any information about their informal caregivers and when that person is unable to provide sufficient information. This is because while there are already guidelines in place for hospitals regarding patients without relatives [21], there is no consensus regarding those without information as to whether they have any relatives.

The limitations of this study are the small number of participants and the fact that some people were not approached, even in the CBPR scheme. Future studies should include more participants from different areas. The strength of this study is that it presents a new classification for caregivers, considering that families are becoming smaller and the retirement age is increasing.

## Conclusion

The conventional approach to contacting informal caregivers is to approach them at places where they generally gather, such as family caregivers’ associations and medical institutions. However, in clinical practice, sometimes we must approach informal caregivers from the starting point—older persons—to discuss future caregiving activities. The practical knowledge that this study imparts is that healthcare professionals will face three stages in reaching informal caregivers: (1) the household form, (2) caregivers’ accessibility, (3) caregivers’ perception of caregiving. In the second tier, some informal caregivers may be inaccessible or unresponsive. In the third tier, there may not be reciprocal awareness of caregiving in some cases. Thus, healthcare professionals should be aware that some informal caregivers are invisible, and they must try hard to reach them.

### Electronic supplementary material

Below is the link to the electronic supplementary material.

Supplementary Material 1

## Data Availability

The datasets used during the current study are available from the corresponding author on reasonable request.

## Abbreviations

LTCI: Long-term care insurance
CBPR: Community-based participatory research
