# Supplementary material for: Inaccessibility, unresponsiveness, inconsistency, and invisibility of informal caregivers of older persons with cognitive impairment: community-based participatory research
Source: BMC Geriatr. 2023 Dec 6;23:812. doi: 10.1186/s12877-023-04542-5 (PMC10702127; doi:10.1186/s12877-023-04542-5)
Supplement: Supplementary file 1 — Supplementary Material 1 [file 12877_2023_4542_MOESM1_ESM.docx]

Additional file. Question examples used in this study.

**Questions in the questionnaire for the older persons;**

Please respond to each item by marking one box per row;

All of the time/Most of the time/More than half the time/Less than half the time/Some of the time/At no time

1. I have felt cheerful in good spirits.
2. I have felt calm and relaxed.
3. I have felt active and vigorous.
4. I woke up feeling fresh and rested.
5. My daily life has been filled with things that interest me.

Do you have any help or assistance from your family or relatives in your current life?

Is there anything else in your current life that you would like more help for?

**Questions in the questionnaire for the caregivers;**

Please tell us about your relationship with the person you care for.

Are you living together with the person you care for? (Yes/No)

How long have you been caring? (< 1 year/1 year to 3 years/3 years to 5 years/3 years to 5 years/> 10 years/Others)

How often do you care? (< one day/month/< one day per week/2 days to 6 days per week/Every day)

Is the person you care for certified in LTCI? (No certification/Support needy 1/Support needy 2/Care needy 1/Care needy 2/Care needy 3/Care needy４/Care needy 5/DK)

Please tell me your age. ( ___years old)

Please tell me your sex. (male/female)

Which of the following applies to your economic status? (Difficulty/Somewhat difficulty/Normal/Somewhat affluent/Affluent)

Which of the following applies to your job? (Full-time job/Part-time job/Quitted due to caregiving/Quitted due to other reason/Not employed)

Do you belong to family caregivers’ association? (Belonging at present/Belonged in past/No belonging)

Do you have someone whom they can consult? (yes/no)

Do you have someone who praise them? (yes/no)

Do you have someone who take them to hospital when sick? (yes/no)

Do you have someone who support their daily lives? (yes/no)
